# Candidemia: An Update on Epidemiology, Risk Factors, Diagnosis, Susceptibility, and Treatment

**DOI:** 10.3390/pathogens14080806

**Published:** 2025-08-14

**Authors:** Juan Pablo Cabrera-Guerrero, Eduardo García-Salazar, Graciela Hernandez Silva, Alberto Chinney Herrera, Erick Martínez-Herrera, Rodolfo Pinto-Almazán, María Guadalupe Frías-De-León, Carlos Alberto Castro-Fuentes

**Affiliations:** 1 Servicio de Microbiología, Instituto Nacional de Enfermedades Respiratorias Ismael Cosío Villegas (INER), Calz. de Tlalpan 4502, Belisario Domínguez Secc 16, Tlalpan, Mexico City 14080, Mexico; jpcguerrero@gmail.com; 2 Servicio de Infectología, Hospital Regional de Alta Especialidad de Ixtapaluca (HRAEI), Servicios de Salud del Instituto Mexicano de Seguro Social para el Bienestar (IMSS-BIENESTAR), Carretera Federal México-Puebla Km 34.5, Ixtapaluca 56530, Mexico; 3 Laboratorio de Micología Molecular, Unidad de Investigación Biomédica, Hospital Regional de Alta Especialidad de Ixtapaluca (HRAEI), Servicios de Salud del Instituto Mexicano de Seguro Social para el Bienestar (IMSS-BIENESTAR), Carretera Federal México-Puebla Km 34.5, Ixtapaluca 56530, Mexico; eduardogs_01@hotmail.com; 4 Servicio de Infectología, Instituto Nacional de Enfermedades Respiratorias Ismael Cosío Villegas (INER), Calz. de Tlalpan 4502, Belisario Domínguez Secc 16, Tlalpan, Mexico City 14080, Mexico; graciela.hernandez.silva@gmail.com; 5 Centro Nacional de Prevención y Control de Enfermedades (CENAPRECE), Francisco de P. Miranda No. 157, Col. Unidad Lomas de Plateros, Álvaro Obregón, Mexico City 01480, Mexico; a.chinney.h@gmail.com; 6 Sección de Estudios de Posgrado e Investigación, Escuela Superior de Medicina, Instituto Politécnico Nacional, Plan de San Luis y Díaz Mirón, Mexico City 11340, Mexico; erickmartinez_69@hotmail.com (E.M.-H.); rodolfopintoalmazan@gmail.com (R.P.-A.); 7 Fundación Vithas, Grupo Hospitalario Vithas, 28043 Madrid, Spain; 8 Efficiency, Quality, and Costs in Health Services Research Group (EFISALUD), Galicia Sur Health Research Institute (IISGS), Servizo Galego de Saúde-Universidade de Vigo (UVIGO), 36213 Vigo, Spain; 9 Unidad de Investigación, Hospital Regional de Alta Especialidad de Ixtapaluca (HRAEI), Servicios de Salud del Instituto Mexicano de Seguro Social Para el Bienestar (IMSS-BIENESTAR), Carretera Federal Mexico-Puebla Km 34.5, Ixtapaluca 56530, Mexico; 10 Posgrado en Ciencias Biológicas, Facultad de Medicina, Universidad Nacional Autónoma de Mexico, Avenida Universidad 3000, Ciudad Universitaria, Coyoacán, Mexico City 04510, Mexico

**Keywords:** *Candida*, *Candida* bloodstream infection, epidemiology, risk factors, diagnosis, antifungal susceptibility

## Abstract

Candidemia is a highly prevalent invasive fungal infection caused primarily by *C. albicans*, *C. parapsilosis*, *C. glabrata* (currently *Nakaseomyces glabratus*), *C. tropicalis*, and *C. krusei* (currently *Pichia kudriavzevii*). Risk factors for the development of candidemia include steroid-induced immunosuppression used in solid organ or hematopoietic transplantation, and neutropenia secondary to infectious or tumorous processes. Alterations in the gut microbiota in people living with HIV, caused by antiretroviral therapy, increase the possibility of colonization by *C. albicans*. Likewise, the presence of a central venous catheter, parenteral nutrition, and abdominal surgery stand out as the main risk factors for the development of candidemia. New diagnostic tools have been developed for the diagnosis of this mycosis that allow the identification of the main species, from improvements in conventional stains such as calcofluor white, which increases sensitivity, as well as technologies such as T2 Candida, MoiM assay, biomarker panel (1,3 β-D-glucan, C-reactive protein, presepsin, and procalcitonin), and, more recently, the development of biosensors for the identification of *Candida* spp. Regarding treatment, the use of micafungin and anidulafungin in patients with obesity defined by a BMI > 30 kg/m^2^ has shown higher survival rates and therapeutic success. Meanwhile, newer antifungals such as rezafungin and fosmanogepix have demonstrated excellent results in the treatment of these patients. Therefore, this review aims to update the epidemiology and risk factors of candidemia, as well as analyze the diagnostic tools and treatments currently available.

## 1. Introduction

Invasive candidiasis (IC) encompasses a group of infectious syndromes caused by *Candida* spp., with candidemia being the most recognized. The latter is defined as the presence of *Candida* in the blood, when identified in at least one positive blood culture in a peripheral or central blood sample [1]. The main etiological agents are *Candida albicans*, *Candida glabrata* (currently *Nakaseomyces glabratus*), *Candida parapsilosis*, *Candida krusei* (currently *Pichia kudriavzevii*), *Candida* (currently *Candidozyma*) *auris*, and *Candida tropicalis* [2].

Because *Candida* spp. is part of the human microbiota, most cases of infection by this fungal genus originate in the endogenous human reservoir, which is altered when the host’s defenses are altered. Thus, *Candida* spp. can cause invasive infection, especially in visceral organs, the vasculature, bones and joints, eyes, and the central nervous system [3]. The most vulnerable patient groups are those with some malignancy and/or immunosuppression, including hematological malignancy, solid organ malignancy, hematopoietic stem cell and solid organ transplantation, recent surgeries, hemodialysis, and malignant wounds caused by the infiltration of skin tissue by tumor cells [4]. This is mainly due to the presence of a central venous catheter and parenteral nutrition conditioned by their immunological status, and the presence of biofilms in the bloodstream, which prevents successful treatment and leads to a high mortality rate [5]. It is worth mentioning that candidemia is a global health problem, especially in the United States, Europe, Asia, and Latin America, with a mortality rate of 36–60% [5].

Despite the extensive information on candidemia, the population’s risk factors are diverse, so those related to the patient’s condition and those related to hospital care must be considered. Meanwhile, in various hospital centers, particularly in developing countries, rapid and reliable tools are lacking. Thus, the diagnosis often proves to be nonspecific and unsuccessful in identifying *Candida* spp., leading to a delay in appropriate antifungal treatment. Therefore, it is necessary to stay up-to-date regarding the current prevalence of this invasive fungal infection, novel antifungal therapies, immunological risk factors and, in particular, the diagnostic tools that allow a prompt and effective approach, due to resistance to antifungals, as well as the increase in cases due to other species of *Candida* that are gaining medical importance such as *C. vulturna* (currently *Candidozyma vulturna*) [6]. Therefore, in this review, we present an update on these topics, with a particular and novel focus on risk factors and treatment in clinical practice.

## 2. Main Etiological Agents of Candidemia

**Figure 1:**
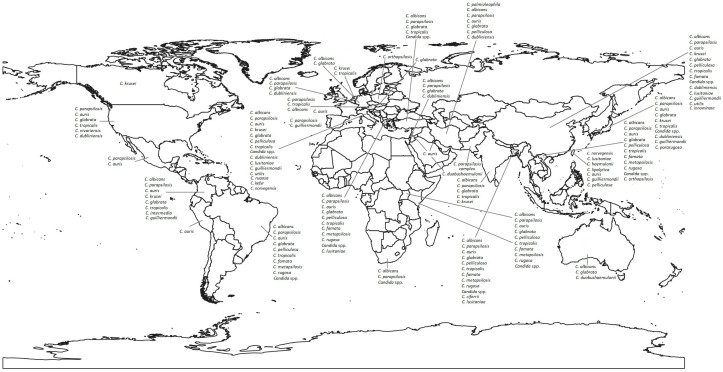
Global distribution of *Candida* species in cases of candidemia reported in the period 2020–2025.

The etiology of candidemia reported in the last five years has been diverse, although most reports come from Asia, followed by Europe, America, Africa, and Oceania (Figure 1). *C. albicans*, *C. parapsilosis*, *C. glabrata* (currently *Nakaseomyces glabratus*), *C. tropicalis*, and *C. krusei* (currently *Pichia kudriavzevii*) are the five most frequent species (Table S1), but the predominant agent remains *C. albicans* [7,8,9,10,11,12,13,14,15,16,17]. In some regions of Bangladesh, Turkey, South Africa, Taiwan, the USA, Israel, Belgium, Spain, and India, non-*albicans Candida* (NAC) species, mainly *C. parapsilosis*, *C. glabrata*, *C. tropicalis* or *C. guilliermondii* (currently *Meyerozyma guilliermondii*), are observed to occupy the first place and *C. albicans* the second or third place in frequency [18,19,20,21,22].

The distribution of species varies depending on age, underlying disease, or geographic region. *C. glabrata* is common in North America, whereas *C. parapsilosis* is more frequent in Latin America, Europe, and Africa [7,8,9,15,19,23,24,25]. A relatively higher incidence of *C. glabrata* and *C. tropicalis* is present in countries in Asia and Oceania. Furthermore, *C. parapsilosis* is particularly common in newborns and is associated with outbreaks of nosocomial infection [7,23], and *C. glabrata* commonly affects the adult population [25,26,27]

Among the NACs that cause candidemia, the presence of some rare or emerging species stands out, such as *C. palmioleophilia*, *C. pelliculosa*, *C. rugosa*, *C. lusitaniae* (*Clavispora lusitaniae*), *C. digboiensis*, *C. utilis*, *C. innominate*, *C. ciferri*, *C. duobushaemulonii*, *C. kefyr*, *C. norvegensis*, *C. intermedia*, *C. pararugosa* (*W. pararugosa*), and *C. auris*. Furthermore, the discovery of species within the *C. glabrata* and *C. parapsilosis* complexes stands out, such as *C. metapsilosis*, *C. orthopsilosis*, and *C. nivariensis* (currently *Nakaseomyces nivariensis*). Infections caused by these species have been reported in patients vulnerable to other complications, such as the pediatric population, patients with severe COVID-19, and those in intensive care [7,8,9,11,12,13,22,24,28,29,30,31,32,33,34,35,36,37,38,39,40,41,42,43,44,45,46,47]. It is worth noting that within NACs, *C. auris* is one of the species with the greatest global distribution. As can be seen, in the last five years it has been reported mainly in Asia, America, Europe, and Africa, while no reports have been published in Oceania (Table S1). *C. auris* is a yeast of priority health importance because of different factors: it is associated with nosocomial outbreaks where patients can remain colonized for long periods of time, and the yeast can also persist on abiotic surfaces due to the low effectiveness of some disinfectants and its ability to form biofilms. Another important factor is the high mortality rate in invasive infections associated with its antifungal resistance, as well as the difficulty in its correct identification [48].

In some studies, yeasts, especially those from NAC, are not identified and are reported only at the genus or complex level [8,10,13,14,19,21,49,50,51]. The lack of species identification at the time of diagnosis of candidemia is a major problem, as the severity of infection varies significantly depending on the species. Although *C. albicans* remains the most common species and does not always cause severe disease, other species that are highly resistant to antifungals may have higher mortality rates, such as *C. auris*, *C. tropicalis*, *C. glabrata*, *C. krusei*, and *C. parapsilosis*, which are associated with medical devices and are therefore more difficult to eradicate [52].

Otherwise, recently reported cases of candidemia within a period of no more than five years were caused by a single species of *Candida*. However, there is at least one report in which two species, *C. albicans* and *C. glabrata* in this case, were involved in the infection of an elderly adult in critical clinical conditions because of necrotizing pancreatitis [27].

It is important to mention that the variation in the distribution of species causing candidemia varies between geographical regions and this depends on genetic and environmental factors, the type of population, age, the use of central venous catheters, and the underlying condition, as well as the type of antifungals used [53]. Changing trends in epidemiology and antifungal susceptibility patterns of six bloodstream *Candida* species isolates over a 12-year period in Kuwait. Thus, the efficient transmission of *C. albicans* and *C. parapsilosis* from one patient to another may contribute to the high number of cases of candidemia caused by these two species [54].

## 3. *Candida* spp. Susceptibility to Antifungals

Of the studies that published antifungal susceptibility testing results for *Candida* spp. causing candidemia, 69% (46/67) showed resistance to one or more antifungal types.

In the last five years, antifungal resistance in cases of *Candida* fungemia has been reported mainly in Asia, followed by Europe, the Americas, Africa, and Oceania. In Asia, the region with the highest number of resistance reports is the east, followed by the west and south, while in Europe, the southern regions have the highest number of resistance reports, followed by the west and east. Regarding the American continent, the northern region reports more resistance than Latin America; in Oceania, only two reports were found. Global data show widespread resistance of *Candida* spp. to fluconazole [11] (Table S1). Resistance to other azoles and other types of antifungals has increased significantly, with variations depending on the geographic region and the species.

### 3.1. Asia

In South Asia, relatively low resistance rates (<14%) have been reported to azoles, echinocandins, amphotericin B, and 5-FC. For voriconazole, posaconazole, and itraconazole, the resistance rate is <5%, with *C. albicans*, *C. parapsilosis*, *C. auris*, *C. lusitaniae*, and *C. glabrata* being the species in which it has been reported most frequently [17,22]. Regarding echinocandins, resistance to caspofungin occurs in *C. albicans*, while *C. glabrata* and *C. parapsilosis* have shown resistance to all three echinocandins [17,55]. High minimum inhibitory concentration (MINC) values of amphotericin B have been detected in isolates of *C. glabrata*, *C. tropicalis*, *C. albicans*, *C. parapsilosis*, and *C. krusei* [17,55,56]. Resistance to 5-FC has only been reported in *C. glabrata* and *C. parapsilosis* [17,56].

In East Asia, resistance rates to voriconazole, posaconazole, and itraconazole vary between <6–42.9% in species such as *C. albicans*, *C. glabrata*, *C. parapsilosis*, and *C. tropicalis*; however, *C. tropicalis* is the species with the highest tendency to present resistance to azoles [12,14,49,50]. Resistance to all three echinocandins has been reported in *C. haemulonii*, but *C. glabrata* and *C. auris* have also shown resistance to micafungin and caspofungin, respectively, with rates of up to 17.8% [14,20,43]. The resistance rate to amphotericin B has been reported to be <6%, mainly in *C. glabrata*, *C. tropicalis*, *C. albicans*, *C. parapsilosis*, *C. krusei*, *C. auris*, and *C. haemulonii* [14,20,43,49,50]. Resistance to 5-FC is rare in this region of Asia, with a rate of up to 0.5% reported in *C. glabrata*, *C. tropicalis*, *C. albicans*, *C. parapsilosis*, *C. krusei*, and *C. haemulonii* [20,49].

In western Asia, resistance to azoles, particularly voriconazole, stands out with rates of up to 13.6, 20, and 50% in *C. albicans*, *C. parapsilosis*, and *C. tropicalis*, respectively [21,57]. Reports of resistance to echinocandins and amphotericin B were scarce; only *C. auris* showed resistance to anidulafungin [47], while *C. doubushaemulonii* and *C. glabrata* showed resistance to amphotericin B [45,58]. No reports of resistance to 5-FC were found.

### 3.2. Europe

In Southern Europe, resistance to itraconazole, voriconazole, and posaconazole has been reported, with *C. tropicalis* presenting the highest rate (32%), along with *C. auris* [9,10,34,35,59]. Resistance to caspofungin, anidulafungin, and micafungin has been reported in several species, including *C. auris*, *C. parapsilosis*, *C. glabrata*, and *C. albicans* [9,10,34,60,61]. Resistance to amphotericin B and 5-FC was rare, with *C. auris* and *C. lusitaniae* exhibiting this phenotype [35,36,38].

In Western Europe, resistance to itraconazole and voriconazole, as well as amphotericin B, was reported only in *C. tropicalis* [62]. No resistance to 5-FC was reported.

In Eastern Europe, no resistance to azoles other than fluconazole, nor to echinocandins, polyenes, or 5-FC was reported [62].

### 3.3. America

In North America, resistance to itraconazole, voriconazole, and posaconazole was observed in *C. auris*, *C. krusei*, and *C. tropicalis* [24,29,63,64]. Resistance to caspofungin and anidulafungin was found in *C. auris* and *C. glabrata*, respectively, while resistance to amphotericin B was found in *C. auris*, *C. albicans,* and *C. parapsilosis* [18,24]. Resistance to 5-FC was observed only in *C. auris* [24,31]

In Latin America, in addition to fluconazole resistance, resistance to amphotericin B has only been reported in *C. lusitaniae* and *C. parapsilosis* [7,23,28,65].

### 3.4. Africa

In Africa, a 31% resistance rate to voriconazole was found in *C. auris*, as well as a 7% rate to amphotericin B in *C. parapsilosis* and a 15% rate in *C. auris* [8].

### 3.5. Oceania

No reports of resistance to any antifungal other than fluconazole were found [26,33]. With this information, we cannot affirm that resistance is higher in Asia and Europe than in the Americas, Africa, or Oceania, since the number of reports found on antifungal resistance in *Candida* spp. causing candidemia was not the same. There were more reports in Asia, while only two in Africa and Oceania. However, what can be observed is a trend towards greater resistance to azoles in *C. albicans*, *C. glabrata*, *C. parapsilosis*, *C. auris*, and *C. tropicalis*, particularly in the latter species. Another important observation is that, given the lack of response to azoles, the use of echinocandins and polyenes has been increasing, leading to the emergence of species resistant to this type of antifungal, as reported in Asia, Europe, America, and Africa. The species in which resistance to echinocandins and amphotericin B stands out are *C. auris*, followed by *C. parapsilosis*, *C. glabrata*, *C. tropicalis*, and *C. albicans*, as well as rare species such as *C. haemulonii*, *C. duobushaemulonii*, and *C. lusitaniae*. Regarding 5-FC, although effective against *Candida* spp., the rapid development of resistance is a problem, as observed in *C. albicans*, *C. tropicalis*, *C. parapsilosis*, *C. glabrata*, *C. krusei*, *C. lusitaniae*, *C. haemulonii*, and *C. auris*. This highlights the growing need for the correct identification of the causative agent and antifungal susceptibility testing for the appropriate management of patients with candidemia.

## 4. Mechanisms of Antifungal Resistance

Different types of antifungals are used in both the prophylaxis and treatment of candidemia, such as azoles, echinocandins, and polyenes, which act on different fungal biosynthetic pathways. Azoles act on ergosterol biosynthesis, echinocandins on cell wall biosynthesis, and polyenes increase membrane permeability by binding to ergosterol. Although azoles, polyenes, and echinocandins are effective against *Candida* species, in recent years many species have developed resistance to one or more types of antifungals. This phenomenon has significant clinical implications because it causes therapeutic failure, posing a threat to patients with *Candida* bloodstream infections worldwide. *Candida* resistance to antifungals occurs through different mechanisms, as described in Table 1. Knowledge and understanding of the mechanisms of antifungal resistance in *Candida* spp. should drive the development of assays that allow for the timely identification of the etiological agent and the presence of resistance in order to contribute to the selection of an appropriate treatment that positively impacts the prognosis of patients with candidemia and other *Candida* spp. infections.

## 5. Risk Factors for Candidemia

The development of candidemia is multifactorial, so two large groups must be considered: factors associated with the host and those related to hospital care.

### 5.1. Host-Related Factors

#### 5.1.1. Immunosuppression and Microbiota

Among the most common scenarios for the presentation of candidemia are steroid-induced immunosuppression, drugs associated with solid organ transplantation, hematopoietic transplantation, and neutropenia secondary to infectious or tumoral processes [71,72]. In these cases, the risk is determined through an adequate clinical history. However, candidemia in apparently immunocompetent patients interestingly reveals that immunological vulnerability ranges from colonization control to the ability to resolve a local or disseminated infection.

The immune response against *Candida* is mainly mediated by Th1 and Th7 responses [72]. Mutations in genes such as *STAT1* and *RORC* can prevent the differentiation of these cells and the production of IL-17 in mucosal tissues, compromising barrier immunity [73,74,75]. Likewise, alterations in the phenotype of costimulatory molecules, such as decreased CD28 and an increase in immune exhaustion markers PD-1/PD-L1 [71], have been associated with disseminated forms such as candidemia and mucocutaneous candidiasis. In the case of HIV, the specific suppression of CD4 not only implies a deficient immune response against *Candida* but also an alteration in the composition of the microbiota and an increase in microbial translocation, because of the loss of mucosal immunity [76]. In vivo studies have demonstrated the important role of bacteria of the genus *Clostridia* in the large intestine, which promote the activation of PPAR-γ receptors through butyrate for the maintenance of physiological hypoxia, and in turn, this reduces the favorable microenvironment for colonization by *Candida* and bacteria of the *Enterobacteriaceae* family [77]. In mice models, the prominent reduction in *Clostridia* abundance creates a favorable microenvironment for *Candida* colonization. Moreover, the authors describe a molecular mechanism involving the disruption of physiological hypoxic environment in intestine. This article was included because it provides a specific mechanism, previously observed in studies not included in the review. In one of them, *Clostridium* species inhibit *Candida* through the production of short-chain fatty acids (SCFAs) [78]. Furthermore, another study, reported than in preterm infants, a reduction in *Clostridium* abundance due to antibiotic use was associated with the development of invasive fungal infection, suggesting an effect that extends beyond colonization [79].

Regarding the humoral adaptive response, the presence of antibodies against the microorganism in patients with candidemia seems to be related to better survival [80]. However, together, genetic factors, the composition and function of the microbiota, and pharmacological interventions make up a spectrum of risks that, although not always evident, should be considered when evaluating susceptibility to candidemia.

Transplantation represents a particularly complex scenario since conditions such as pharmacological immunosuppression, a torpid immune response, the use of antibiotics and prolonged use of catheters, as well as recurrent surgical procedures, come together [81]. The approximate time in which there is a greater risk for candidemia varies with the type of transplant; in the case of kidney, lung, heart, hematopoietic cells, and pancreas, the risk period for candidemia ranges from the first month to 3.5 months post-transplant, generally attributable to immunosuppressive therapy in combination with steroids and others such as tacrolimus or belatacept [82].

#### 5.1.2. Chronic Diseases and Age

Age is a determining factor in the risk of candidemia. In childhood, congenital genetic defects, both immunological and those associated with neoplasia, predispose to severe infections and malnutrition with high mortality rates at early ages [83]. Furthermore, immunological memory, which is still developing, can generate a slower and less effective response to *Candida* colonization or infection. Among the high-risk pediatric groups, those with acute lymphoblastic leukemia stand out, where the incidence of candidemia can reach up to 70% [84]. Another vulnerable group is children who require prolonged use of central venous catheters.

In contrast, in adults, the accumulation of genetic defects results in metabolic disorders (dyslipidemia, diabetes) [85,86] and the aging of the immune system. This functional impairment of the immune system reduces the effectiveness of responses to new pathogens, including opportunistic fungi [87].

In this population group, although risk factors are shared, prolonged hospitalization and previous exposure to both antibiotics and antifungals should be considered, which limits therapeutic options for the early and effective treatment of candidemia [88]

### 5.2. Factors Related to Hospital Care/Intensive Care Unit

#### 5.2.1. Health Care

Progressive colonization by *Candida* in hospitalized patients, particularly those in intensive care units (ICUs), has been extensively documented as a relevant factor in the pathogenesis of candidemia. A multicenter prospective study demonstrated a significant increase in cutaneous colonization by *Candida*, rising from 27.3% at hospital admission to 52.7% by the eighth day of hospitalization. Moreover, the ICU environment was identified as an independent factor associated with colonization (odds ratio [OR] = 2.03; 95% confidence interval [CI]: 1.22–3.39), highlighting the role of the hospital microbiome as a key determinant in dysbiosis and the risk of invasive fungal infection [89].

This trend reflects the cumulative impact of hospital exposure, and frequent invasive interventions in the ICU disrupt the microbiota and promote the proliferation of opportunistic fungi.

#### 5.2.2. Use of Broad-Spectrum Antibiotics

Intensive care unit stays most often result in the use of broad-spectrum antimicrobials [90]. The use of antibiotics increases the possibility of *Candida* colonization in the gastrointestinal tract, generating dysbiosis and affecting the interaction with the bacterial microbiome. In this sense, an OR of up to 5.61 (1.82; 4.1) has been documented for the development of candidemia due to the use of broad-spectrum antimicrobials for more than 72 h in critically ill patients [91]. Recently, the risk of developing candidemia has been compared between different antimicrobial groups, where carbapenems were associated with a higher risk of candidemia compared to other beta-lactams [92].

#### 5.2.3. Central Venous Catheter

Central venous access is among the most used intravascular devices in hospital settings; however, their use carries risks, particularly bloodstream infections. The initial adhesion capacity of *Candida* to the surface of the central venous catheter is considered an important virulence factor, in addition to the subsequent formation of biofilm, which leads to lower susceptibility to antimicrobials [93]. Some studies have shown that the risk of infection increases with the number of days the catheter is in place (≥14 days) and the presence of more than one lumen [94].

#### 5.2.4. Total Parenteral Nutrition

Parenteral nutrition provides hydration and nutrients to critically ill patients or those intolerant to enteral nutrition. These formulas contain water, dextrose, essential amino acids, electrolytes, and a lipid emulsion. The fatty acids contained in the lipid emulsion may contribute to the growth and biofilm formation of species of *Candida* [91].

#### 5.2.5. Development of Biofilms

Different *Candida* species exhibit distinct characteristics in biofilm formation, interestingly not only depending on the species but also on the type of model, i.e., in vitro, in vivo, or in patients. For instance, *C. glabrata* produces biofilm in vitro faster than others; however, in vivo, *C. glabrata* most often colonizes mucous and its adhesion to epithelia is low. Another interesting case is *C. auris*; although its pathogenicity and virulence are high, its biofilm is unstructured and is thus able to contribute to its high resistance to antifungal treatments. Even though this was outlined in a specific review of biofilm formation [95], for the purpose of this work, it is essential to highlight that *Candida* biofilms confer protection against both immune response and antifungal agents. In addition, we can use *C. albicans* as example to explain the biofilm formation, which follows the sequence below.

Adhesion: blastospores adhere to both biotic and abiotic surfaces (including catheter surfaces, among others) through the action of adhesion proteins such as Agglutinin-Like Sequence (ALS) and Hyphal Wall Protein 1 (HWP1). Proliferation: cells form a layer through an active division and begin the transition from yeast to hyphae and pseudohifas. Maturation: the production of extracellular matrix with β-glucans, proteins, and extracellular DNA contributes to the three-dimensional formation of the biofilm. Dispersion: daughter cells are released from mature biofilm, ready to colonize other sites [96].

#### 5.2.6. Abdominal Surgery

**Figure 2:**
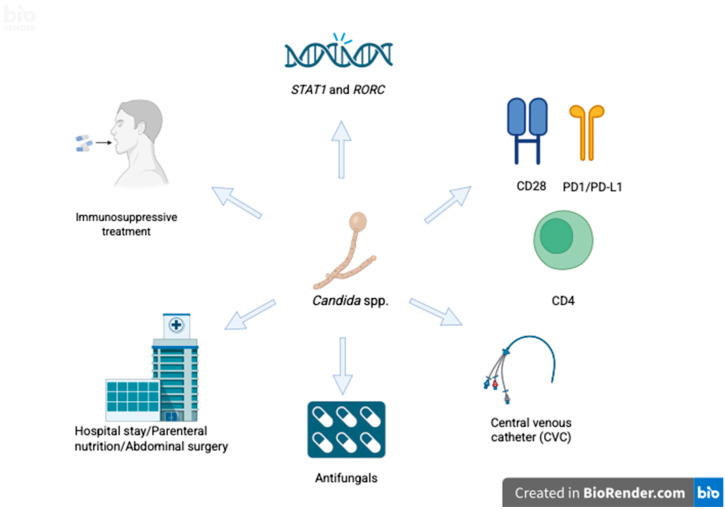
Reported risk factors for the development of candidemia include immunosuppressive therapy associated with solid organ and hematopoietic stem cell transplants, as well as mutations in genes such as *STAT1* and *ROC*, which compromise barrier immunity against *Candida* spp. Decreased CD28 and increased PD-1/PD-L1 markers are also present. In the context of HIV infection, CD4 suppression influences the deficiency of the immune response against *Candida* spp. The use of a central venous catheter is a risk factor for bloodstream infection. Furthermore, surgical intervention, particularly a history of abdominal surgery and parenteral nutrition, facilitates the colonization of *Candida* spp. from the digestive tract to the circulatory system. Finally, the use of antifungals and prolonged hospital stays are factors that contribute to the development of candidemia.

In patients with a history of abdominal surgery presenting with intra-abdominal candidiasis, a 6% prevalence of concomitant candidemia has been reported. The most frequently described procedures are intestinal perforation, anastomotic leaks, liver transplantation, and necrotizing pancreatitis. These procedures facilitate the passage of *Candida* that colonizes the digestive tract into the circulatory system [97]. Meanwhile, the presence of abdominal drains and the risk of *Candida* colonization have recently been described as risk factors for intra-abdominal candidemia and the consequent risk of passage into the bloodstream (Figure 2) [90]. 

## 6. Diagnosis of Candidemia

Currently, the management of fungal infections is limited by the lack of diagnostic tools, which in turn impedes the initiation of antifungal therapy and leads to prolonged hospital stays. Therefore, rapid and reliable diagnostic tools for the diagnosis of candidemia are necessary.

### 6.1. Blood Cultures and Histopathology

Blood cultures are known to be the most useful diagnostic tool in the diagnosis of candidemia, being the “gold standard” according to the European Society for Clinical Microbiology and Infectious Diseases (ESCMID) and the Infectious Diseases Society of America (IDSA) [98]. This diagnostic test is characterized by being quite easy to perform; however, the efficiency of the procedure is limited because *Candida* spp. is isolated in only 21–71% of patients with invasive candidiasis [99]. Despite this, it has been reported that sensitivity can be improved by increasing the sample volume and testing frequency during the suspected diagnosis. However, blood culture is a diagnostic alternative that takes between 72 and 96 h to indicate a result, which delays the establishment of appropriate treatment.

Another limitation of blood cultures is their low yield in newborns, as blood and cerebrospinal fluid are generally sterile. Therefore, when candidemia is suspected in the neonatal population, the use of predictors such as thrombocytopenia and elevated C-reactive protein has been recommended for the diagnosis of candidemia [99]. Furthermore, the role of procalcitonin in the diagnosis of candidemia in high-risk neonatal patients has been evaluated.

In a study involving ninety patients in the Neonatal Intensive Care Unit, six developed invasive candidiasis due to *C. albicans* and *C. parapsilosis*. Two different methods for estimating procalcitonin levels were compared, with no statistically significant differences found between the methods. Four of the six patients had a slight increase in procalcitonin (0.5–1 ng/mL). Only one of the four had a high procalcitonin level; however, this patient also had fungal and bacterial sepsis [100]. Unfortunately, the diagnostic utility of procalcitonin in candidemia could not be established. However, in a review comparing various studies to establish cutoff points between candidemia and bacteremia, the evidence did not support the utility of procalcitonin. Therefore, it should not be considered a differential diagnostic tool because the values reported in the various studies are insufficiently discriminatory to guide therapeutic decisions [101].

To improve the diagnostic performance of blood cultures, the use of special stains has been reported, such as periodic acid–Schiff staining, which can detect polysaccharides and glycoproteins in the fungal cell wall. Gomori–Grocott methenamine silver staining, which stains carbohydrates, is also effective. Additionally, the use of fluorescent brighteners, such as calcofluor white, can increase sensitivity [102].

In a study by Chen et al. [102], the authors reported a modified calcofluor blank assay to stain blood samples from patients with candidemia. The authors developed this stain based on calcofluor (0.05%), Evans blue (0.025%), KOH (10%), antifluorescent decomposer glycerin (10%), diphosphatidylglycerol (0.5%), and ascorbic acid (0.1%). This technique proved to be quick and timesaving, taking the same amount of time as a KOH test (10 min), followed by observation under a fluorescent microscope. Furthermore, since it is a brightener commonly used in the printing and textile industries, it is easily obtained. This modification identified a sensitivity of 90% and a negative predictive value of 82% compared to the traditional culture method (blood culture) and KOH staining. Furthermore, this method reduced processing time, allowing for the rapid detection of fungal agents, from a concentration of 10^5^–10^7^ *C. albicans* cells per mL.

Furthermore, a prospective study including 244 adult and pediatric patients admitted to the intensive care unit determined the time to blood culture positivity. This study demonstrated a statistically significant difference (*p* = 0.04) between the time to peripheral blood culture positivity for several yeast species in pediatric patients. This difference was identified between *C. glabrata* (22.8 h) vs. *W. anomalus* (31.6 h) and *C. jadinii* (18.3 h) vs. *W. anomalus* (31.6 h) in pediatric patients. Conversely, in adult patients, the differences in time to positivity were identified between *C. albicans* (34.55 h) vs. *C. tropicalis* (23.07 h). Specifically, in isolates resistant to amphotericin B, such as *C. parapsilosis*, the average time to culture positivity was 51.7 h, compared to when it showed sensitivity to the same antifungal (34.5 h). For its part, *C. krusei* grew in 17.2 h when it showed resistance to micafungin, while in cases of susceptibility it took 86.6 h to develop [103]. Additionally, the time to positivity in bloodstream infection by *Candida* spp. has been evaluated as a prognosis for mortality.

Keighley et al. 2023 [104] conducted a prospective multicenter study of 527 episodes of candidemia in Australia. The authors determined the time to blood culture positivity with *C. albicans* growth at 49 h, with a day 30 mortality of 28–43%. For the *C. glabrata* complex, the mortality was almost similar (28–46%), but with a longer time to positivity (55 h). Meanwhile, the *C. parapsilosis* complex required 48 h for growth, where mortality was lower (3–16%) compared to *C. albicans* and the *C. glabrata* complex. *C. tropicalis*, on the other hand, reported the shortest time to positivity (27 h), but with a considerable mortality of 24–65%. Finally, *P. kudriavzevii* had a mortality rate of 7–57% and a time to positivity of 33 h. The reported findings suggest that a large inoculum in the bloodstream will result in a shorter period of positivity and a poor outcome. However, it is important to consider that other factors, such as host-intrinsic factors, may influence the outcome.

### 6.2. Molecular-Based Techniques

#### 6.2.1. Polymerase Chain Reaction (PCR)

These types of tests can be performed directly on whole blood, serum, or plasma without depending on the blood culture results. However, they depend on the concentration of the fungus in the blood, so sensitivity can be a limiting factor.

Currently, there is a wide range of tests that are performed based on the extraction of genetic material present in the sample. Among the leading tests on the market are AurisID^®^ and Fungiplex^®^ *C. auris* (currently *Candidozyma auris*) based on qPCR and real-time PCR, respectively. Both tests identify *C. auris* [105]. CandID^®^, Fungiplex^®^ Candida, Fungiplex^®^ Universal, MycoReal Candida, and MagicplexTM Sepsis, based on Multiplex PCR, have the capacity to identify *C. albicans*, *C. glabrata*, *C. parapsilosis*, *C. krusei*, *C. dubliniensis*, and *C. tropicalis*. Finally, tests such as Hybcell Pathogens DNA xB, SepsiTestTM-UMD, and MycoReal Fungi are microarrays and are focused on sequencing the 28S, 18S, and ITS regions [106]. It is worth mentioning that despite being rapid assays that allow the establishment of prompt antifungal therapy, many of these have not yet been validated for the diagnosis of invasive candidiasis in multicenter cohorts, which calls into question their diagnostic capacity.

Another highly useful tool in the diagnosis of candidemia is the RAPD-PCR technique, characterized by its ease and low cost. A total of 39 *C. albicans* strains isolated from different hospitalized patients were analyzed using microsatellite analysis using RAPD-PCR, which yielded between 10 and 11 polymorphic profiles. Two markers were developed (CDC3 and HIS3), allowing the identification of six and seven alleles, respectively. The authors identified a high discriminatory power with the implementation of this technique. Analysis using RAPD-PCR and microsatellites revealed similarities between *C. albicans* isolates. Likewise, groups of several genotypes were formed, confirming their similarity among isolates [105]. This technique has allowed the identification of *C. parapsilosis* strains from polymorphic profiles in approximately 4 h and is an accessible technique, due to the requirement of end-point PCR equipment [107].

#### 6.2.2. Next-Generation Sequencing

ITS regions and/or the D1-D2 domain are the most widely used method for routine fungal identification in laboratories. This is despite their limitations, particularly the inability to differentiate closely related species. Furthermore, performing this type of methodology is time-consuming and requires highly qualified personnel [99]. However, this methodology has been used to evaluate echinocandin resistance in *C. parapsilosis*.

Currently, fluconazole-resistant *C. parapsilosis* (FLCR-Cp) has caused persistent outbreaks, even in patients who have not been exposed to azoles. Although echinocandins represent a therapeutic alternative, multidrug-resistant *C. parapsilosis* (MDR-Cp) isolates have been isolated from patients in various parts of the world [108]. In the context of multidrug resistance due to *C. parapsilosis*, whole genome sequencing represents another diagnostic tool. In addition to being able to evaluate the clonality of the isolates, CRISPER-Cas9 has been used to evaluate the echinocandin resistance of this species due to the *FKS1^R658G^* gene in a murine model of *Galleria mellonella*. Thus, whole genome sequencing not only allows for species-level identification but also contributes to establishing infection control by evaluating isolates and identifying, through clonality, those isolates causing hospital outbreaks and establishing antifungal treatment strategies [108].

#### 6.2.3. T2 Candida

A novel FDA-approved assay from Biosystems for candidemia has been developed to detect invasive *C. albicans*, *C. tropicalis*, *C. parapsilosis*, *C. krusei*, and *C. glabrata* infection, with a detection limit of one colony-forming unit (CFU)/mL from blood and a mean time of <5 h [109].

The T2 Candida assay has demonstrated modest sensitivity and specificity, however; by combining this assay with biomarkers such as 1,3 β-D-Glucan, intra-abdominal candidiasis has been successfully diagnosed. In the study led by Park et al. [110], the authors evaluated the clinical performance of the T2 Candida assay in 2764 patients at a tertiary care hospital in Seoul, South Korea, between 2022 and 2023. T2 Candida performance was assessed using blood samples obtained from patients on the same day as blood cultures. The T2 Candida system was able to detect three *C. albicans*/*tropicalis*-positive specimens and one *C. krusei*/*glabrata*-positive specimen. However, it failed to detect two specimens of *C. glabrata*. In this study, the *Candida* spp.-negative cases had a positivity time of >60 h. This contrasts with the 11 cases detected by T2 Candida and blood culture with a positivity time of <50 h. Thus, this study demonstrated its acceptable diagnostic performance, with a 95% concordance. It is worth mentioning that the diagnostic performance of T2 Candida is characterized by a sensitivity of 65–100% with a specificity of 90–100%. In addition, it can successfully identify candidemia in patients receiving antifungal treatment [110].

Krifors et al. [111], evaluated the T2 Candida MRI assay in a prospective multicenter study between 2019 and 2021. Of the 134 patients evaluated, 13 were classified with probable intra-abdominal candidiasis. T2 Candida presented a sensitivity of 46%, a specificity of 97%, a positive predictive value of 61%, and a negative predictive value of 94%, being superior to the biomarker 1,3 β-D-glucan. All positive T2 Candida results were consistent with blood culture results. Additionally, Camp et al. [112] evaluated the T2 Candida MRI panel compared to blood culture and Septifast for the detection of five species of *Candida* in high-risk patients with suspected Candidemia. Twenty-two samples from seventeen patients were classified as probable candidemia. The sensitivity of T2 Candida was 63.6% in cases of proven candidemia. For proven and probable candidemia, the sensitivity of T2 Candida was 73.0%.

To date, T2 Candida represents a rapid and accurate non-culture-based diagnostic tool using blood for the diagnosis of Candidemia. The high negative predictive value of T2 Candida makes it a valuable tool for physicians to base therapeutic decisions on. However, it is limited to the identification of only five species of *Candida*, in addition to low sensitivity in populations with a low prevalence of invasive candidiasis [109].

#### 6.2.4. New Molecular Tools

Park et al. [113] evaluated the diagnostic capacity of a novel assay designed for the detection of *Candida* spp. from positive blood cultures. Thirty-nine cases of candidemia and forty cases of bloodstream infection were included in the study as negative controls. Species were identified by MALDI-TOF, which was considered the reference standard. Positive percentage concordance and negative percentage concordance were evaluated. The MoiM assay demonstrated a positive percentage concordance of 97.1% and a negative percentage concordance of 100% for the detection of *C. albicans*, *C. glabrata*, *C. tropicalis*, *C. parapsilosis*, and *C. krusei*. This novel assay also adequately discriminated *C. guilliermondii* and *C. lusitaniae*.

#### 6.2.5. Matrix-Assisted Laser Desorption–Ionization Time-of-Flight (MALDI-TOF)

This technique compares the species-specific spectra of the analyzed fungi with a mass spectral database. This results in rapid and accurate identification, with the analysis taking only 15 min. Furthermore, unlike other techniques, it does not require expensive reagents or highly qualified equipment. However, the cost of the equipment is a major limitation for laboratories in developing countries [99].

In a study by Balaji et al. [114], the authors evaluated the time to positivity in bloodstream infections caused by *Candida* spp., as well as the impact on patient outcome. Positive Candida cultures were identified using MALDI-TOF. *Candida auris* (currently *Candidozyma auris*) accounted for 28% of the isolates, with *C. tropicalis* being the second most common etiological agent isolated in the study population, followed by *C. glabrata* and *C. parapsilosis*. Other species identified were *C. guilliermondii* and *C. orthopsilosis*. MALDI-TOF has also been successfully used to identify *C. krusei* [16,115].

Likewise, the MALDI-TOF technique has been implemented for the identification of *Candida* spp. by performing said identification in blood cultures, thereby reducing the typing time to two days. The authors report results from a rapid methodology for the identification and determination of the susceptibility profile of *Candida* spp. This developed technique was efficient, reproducible, and easy to perform, allowing the patient to receive therapy promptly [116]. Furthermore, MALDI-TOF was the most reliable method for identifying *C. auris* in patients with central line-associated infection, compared to CHROMagar and VITEK. This makes MALDI-TOF a highly useful method for identifying fluconazole-resistant *C. auris* in the intensive care unit [117].

### 6.3. Non-Culture-Based Methods

#### 6.3.1. 1,3 Beta-D-Glucano

Currently, other non-culture-based diagnostic alternatives include the use of 1,3 β-D-glucan (BDG). This is a biomarker for the diagnosis of invasive infections, including invasive candidiasis, and its use in serum is part of the EORTC mycological criteria for the diagnosis of invasive fungal infections [118]. This biomarker has a sensitivity of 92% and a specificity of 81% for the diagnosis of invasive candidiasis, However, the risk of false positives has been evidenced in patients receiving albumin, intravenous immunoglobulin, and systemic antimicrobials [119].

In a study conducted by Zcharioudakis et al. [120], the authors evaluated two methods for the diagnosis of Candidemia in their hospital. First, the combination of blood cultures plus T2 Candida (Procedure 1) and second, in addition to blood cultures and T2 Candida, the use of the biomarker 1,3 β-D-glucan was considered (Procedure 2). Of the 120 patients evaluated with 1,3 β-D-glucan, 29 were positive. A significant difference was observed in the implementation of the nearby antifungal of 40% of practice 1 compared to practice 2, which includes 1,3 β-D-glucan. Likewise, similar rates of antifungal discontinuation were identified from the two procedures. However, the use of 1,3 β-D-Glucan was interpreted variably by physicians and influenced the increase in the use of antifungals without correlating with a clinical benefit for the patient.

On the other hand, Meng et al. [98] developed an assay based on *Candida* anti-mannans IgM and IgG. Anti-mannan is a biomarker derived from the *Candida* cell wall that was implemented to perform the diagnosis of two *Candida* anti-mannan assays. This study comprised 40 patients with Candidemia, 48 patients with *Candida* colonization, 213 patients with possible *Candida* colonization but without infection data, and 55 controls. The sensitivity of the IgM assay was 0.78–0.80, while for the IgG assay it was 0.68–0.75. Meanwhile, the specificities for the IgM and IgG assays were 0.97–0.98 and 0.91–0.94, respectively. The authors reported a better diagnostic performance of the IgM assay compared to IgG. Conversely, combining both assays improved sensitivity, although specificity was reduced.

In addition to 1,3 β-D-glucan, other biomarkers such as C-reactive protein, presepsin, and procalcitonin have been evaluated, and a biomarker panel has been developed in an integrated manner [119]. This biomarker panel has been developed and validated in 165 adult patients for the diagnosis of invasive candidiasis. When evaluating the biomarker characteristics, 1,3 β-D-glucan demonstrated a sensitivity of 96.6%, a specificity of 97.2%, a positive predictive value of 94.9%, and a negative predictive value of 98.1% at a cut-off value of 200 pg/mL (*p* ≤ 0.001), while presepsin had a higher sensitivity and a negative predictive value (100%), as well as a very low positive predictive value (36.5%) and specificity (5.6%) at a cut-off value of 700 pg/mL (*p* ≤ 0.001). Thus, combining presepsin and 1,3 β-D-Glucan improves the diagnostic approach to invasive candidiasis. In particular, the usefulness of the presepsin biomarker was more accurate in predicting mortality on day 28; it is worth mentioning that presepsin levels are significantly reduced after day 14 with antifungal therapy with echinocandins (*p* = 0.0012).

Kinet-Poleur et al. [121] recently evaluated three biomarkers for the diagnosis of Candidemia: CAGTA IgG VirClia Monotest, the Wako β-D-glucan test, and CandId OLM RT-PCR. A total of 35 cases of Candidemia and 20 controls were included. CAGTA IgG VirClia Monotest demonstrated low sensitivity (46%) and moderate specificity (75%), while the Wako β-D-glucan test and CandId OLM RT-PCR exhibited the highest sensitivities (74% and 71%, respectively) and 100% specificity. By combining the Wako β-D-glucan test and CandId OLM RT-PCR, the diagnosis was improved, achieving a sensitivity of 91% and a specificity of 100%. However, a misidentification between *C. albicans* and *C. dubliniensis* was recognized with CandId PCR.

#### 6.3.2. Biosensors for the Identification of *Candida* spp.

A biosensor is defined as an analytical device that translates biological information from chemical reactions into a quantifiable signal proportional to the strength of the reaction. The applicability of these innovative technologies will depend on the sensitivity, selectivity, reproducibility, reusability, detection limit, and response time [122].

Multiple biosensors for the detection of *Candida* spp. have been developed, including electromechanical impedance, piezoelectric immunosensor, optical biosensor, loop-mediated isothermal amplification (LAMP), lateral flow strip, particle-mediated immunosensor, gene sensors, fluorescence and flow cytometry, electromechanical, microfluidic hydrodynamic cell trapping and trapping, and epifluorescence (Table 2). These were developed with the capacity to identify *C. parapsilosis*, *C. albicans*, *C. krusei*, *C. tropicalis*, *C. glabrata*, and *C. auris* from clinical samples, cultures, *Candida* antigens, whole blood, human urine, and target DNA sequencing, with sensitivities from 106 cells/mL to 1 fg [107]. Thus, biosensors offer a fast, accurate, and cost-effective solution for diagnosing *Candida* spp. infections.

## 7. Treatment of Candidemia

Since the last publication of the ESCMID [123] and IDSA [124] guidelines in 2016, which defined recommendations for the diagnosis, treatment, and follow-up of patients with candidemia and invasive candidiasis, mortality from candidemia has been reported to range from 27% to 52.7% [125]. This variation is due to multiple factors such as the presence of neutropenia, patients receiving steroid treatment, infection with certain species of *Candida* [126], the early initiation of appropriate antifungal treatment [127], a high APACHE II score, renal replacement therapy [128], chronic liver disease, neoplastic disease [129], and failure to remove vascular access [130]. Likewise, variable mortality has been reported across geographic regions, hospital centers, and even across care areas within the same hospital [49].

Compliance with these recommendations has a significant impact on clinical outcomes in patients with candidemia. Some studies have evaluated adherence using the EQUAL Candida Score, documenting the degree of adherence as an independent predictor of survival. However, despite a high adherence rate, mortality from this cause remains high, reaching up to 37% [131].

### 7.1. Antifungal Treatment

Objectively considering the impact of candidemia, where high mortality is related to the critical condition of patients and to the microbiological characteristics of the fungus (morphotypic variation, the expression of pathogenicity factors, metabolic flexibility, the evasion of the immune system) [132], the limitations of available diagnostic tests and the fungus–antifungal interaction, particularly in the context of clinical scenarios where susceptible strains are identified and treatment failure is documented, are secondary to phenomena such as tolerance, phenotypic heterogeneity, biofilm formation, and adaptive response to stress [48,69,133]. In this sense, in certain clinical contexts and in the presence of risk factors that pronounce these limitations, evidence supports the benefit of prompt treatment [134].

Currently, various therapeutic strategies have been developed with the aim of optimizing treatment and, consequently, improving clinical outcomes, mainly reducing mortality [135,136]. Therapeutic strategies are focused on the clinical context and microbiological evidence. These include prophylaxis, focused on high-risk patients, identified by clinical-epidemiological risk factors; preemptive therapy, in high-risk patients with microbiological evidence of fungal infection, but without diagnostic confirmation; empirical therapy, which is based on the presence of suggestive clinical manifestations summarily with clinical and epidemiological factors; and targeted treatment, defined by microbiological confirmation by culture, that is, individualized to the susceptibility profile and clinical status of the patient [123].

### 7.2. Profilaxis

Recommendations on the indication of prophylactic therapy are controversial secondary to the heterogeneity in the methodological quality and the risk of bias in the published studies.

### 7.3. Newborn Patients Weighing < 1000 g

*Candida* spp. has been reported as the third cause of late-onset sepsis in neonatal intensive care units, specifically in patients with low birth weight < 1500 g [123,137]. Kaufmann et al. [138] documented, derived from a prospective clinical trial, that prophylactic therapy with fluconazole for six weeks in neonates weighing < 1000 g significantly reduced the incidence of invasive *Candida* infection, with an absolute risk difference of 20% (95% CI: 0.04–0.36; *p* = 0.008). In addition, a significant reduction in multifocal colonization was observed in patients who received prophylaxis (18 vs. 52%; *p* = 0.003), but without impact on mortality, nor change in the phenotypic susceptibility pattern or in the incidence of adverse effects [138]. These findings were confirmed in a systematic review and meta-analysis that included eight clinical trials, reporting a significant reduction in colonization of 68%, with a relative risk of 0.32 (95% CI 0.24–0.42; *p* = 0.00001), as well as a significant reduction of up to 60% in the incidence of invasive infection (RR 0.40; 95% CI 0.22–0.72; *p* = 0.002). Subgroup analysis showed no significant differences between the administration of doses of 3 mg/kg and 6 mg/kg. There was also no significant effect on mortality (RR 0.79; 95% CI: 0.60–1.03; *p* = 0.08, I2 0%), in addition to reporting an adequate safety profile for neurodevelopment [139].

However, an increase in *C. parapsilosis* colonization has been reported, although without significant differences. While these findings are in line with the initial studies supporting the use of prophylactic therapy in this risk group, in which the primary species was *C. albicans*, the current epidemiological landscape, with an increase in the incidence of infections caused by non-albicans species [8,140], presents a scenario that requires evaluation in areas with a high incidence of these species.

### 7.4. Patients with Prolonged Neutropenia

The rate of invasive fungal infections in patients with hematological malignancies has been reported at 1.7 per 100 people per year, with candidemia/invasive candidiasis being the second most common clinical syndrome, up to 37%, surpassed only by invasive aspergillosis (IA) [141]. Studies have been conducted to evaluate the impact of prophylactic treatment in this at-risk population. A multicenter, randomized clinical trial published by Cornely et al. [142] demonstrated that prophylactic treatment with posaconazole is superior to that with fluconazole/itraconazole. This study included patients with prolonged neutropenia who were undergoing chemotherapy for acute myeloid leukemia or myelodysplastic syndrome. In this population, prophylactic treatment with posaconazole demonstrated a decrease in the incidence of confirmed or probable fungal infection (2% compared with 8%), with a risk reduction of −6% (95% CI, −9.7 to −2.5; *p* < 0.001). Additionally, a significant impact was demonstrated in the reduction in mortality at 100 days of follow-up, with 14% mortality in the posaconazole group and 21% in the comparison group (*p* = 0.04).

In this regard, the current recommendation for prophylaxis in patients with prolonged neutropenia with an antifungal agent that targets filamentous fungi, such as posaconazole, is a recommendation with an A1 level of evidence. When analyzing the incidence of fungal infections, the net effect of prophylaxis in reducing invasive fungal infection is clearly significant in the group of patients with IA [142].

When evaluating the impact of prophylactic antifungal therapy, in addition to assessing its effect on reducing the risk of infection, it is necessary to consider the risk of intercurrent fungal infection during its administration. According to some series, the incidence of escape fungal infections is influenced by risk factors, population size, and the type of antifungal administered for prophylaxis [143,144,145]. The identification of *Candida* spp. has been reported among the three main etiologies of intercurrent infection, with an incidence of up to 18%, compared with 40% for *Aspergillus* spp. and 20% for *Mucor* spp. When specifically analyzing the species of *Candida* reported in cases of intercurrent candidemia, the main species reported is *N. glabratus*, but when analyzing by type of antifungal, the main species reported in patients with prophylactic therapy with posaconazole is *C. parapsilosis* compared to voriconazole (2.56% vs. 18%, respectively) [143].

### 7.5. Critically Ill Patients with Abdominal Surgery

In patients with recent abdominal surgery, recurrent gastrointestinal perforation, or anastomotic leak, prophylactic therapy with fluconazole is recommended [146]. Evidence in this patient group is limited by the patient context (type of intervention), severity of illness, and the methodological quality of the studies, most of which are retrospective. In this setting, prophylaxis has been evaluated in patients with liver and pancreas transplants.

The American Society of Transplantation identifies reintervention, hemodialysis, transfusion of >40 units of blood, choledojejunostomy, and *Candida* colonization as risk factors for primary invasive surgical site infection due to *Candida*; therefore, it recommends antifungal prophylaxis for 2–4 weeks after transplantation [147]. In this regard, Carugati et al. [148] retrospectively evaluated 470 liver transplant patients receiving prophylactic therapy with fluconazole, micafungin, and no prophylaxis. The proportion of patients who developed primary invasive surgical site infection during prophylactic treatment with fluconazole was 1%, compared with 9.4% and 1.3% with micafungin and no prophylaxis, respectively. These results contrast with those reported by López-Medrano [149], in patients with pancreatic transplants, where no difference was found in prophylaxis with fluconazole vs. micafungin with a range of invasive candidiasis of 21.7% and 24.2% (*p* = 0.67), respectively.

### 7.6. Anticipatory Therapy

Recently, an area of recent and growing development is proactive therapy in infectious syndromes with high morbidity and mortality. In candidemia and invasive candidiasis in patients with risk factors, the initiation of proactive therapy based on the clinical context has been proposed, specifically due to persistent fever and shock, once other infectious etiologies have been ruled out in addition to a high index of suspicion or some fungal markers [146]. Markers such as 1,3 β-D glucan, mannan antigen, and antibodies against mannan have been evaluated with various approach algorithms [136,150]. Preemptive treatments guided by both clinical context and fungal markers have not shown an impact on mortality, the incidence of subsequent invasive candidiasis, days of hospital stay, or treatment costs, only on a decrease in the days of administration of antifungal therapy (54% vs. 2%). Recently, Erb et al. [151] reported contrasting results when evaluating the termination of antifungal therapy based on an algorithm for determining 1,3 β-D-glucan and mannan antigen in 41 randomized patients. The results did not show a significant difference for any of the evaluated outcomes, including mortality and the administration of antifungals with an algorithm based on the negativity of the markers evaluated at two termination points (day 1 and 2), which are secondary to its low specificity in critically ill patients. In this regard, there is no solid evidence to recommend terminating antifungal therapy in critically ill patients with a high index of suspicion based on the determination of these markers. This calls for the first search for a key population and an algorithm based on specific markers with cost-effective cutoff points.

### 7.7. Therapy

The recommended first-line treatment in severe patients with candidemia without deep candidiasis and without neutropenia are echinocandins, due to evidence of superiority in microbiological eradication and therapeutic success rate, in addition to a favorable safety profile, broad spectrum of susceptibility, low risk of interactions, and low risk of acquired resistance [123,124,146,152,153].

Treatment with echinocandins has been associated with a significant reduction in mortality in patients with candidemia (OR, 0.50; 95% CI, 0.35–0.72; *p* < 0.0001). This was demonstrated by Andes et al. [154] in a systematic review and meta-analysis aimed at evaluating the effect on 30-day mortality, as well as the clinical and microbiological response at the end of treatment in 1915 patients with candidemia and invasive candidiasis treated with azoles, echinocandins, and polyenes. The results showed that patients treated with echinocandins had a lower mortality rate compared with patients treated with other antifungals (27% vs. 36%, *p* < 0.0001), and this effect was present even in severely ill patients (APACHE II score > 24). Furthermore, patients receiving micafungin had a higher survival rate and therapeutic success, while paradoxically, they had higher mortality rates and therapeutic failure rates (30% and 29.6% compared with 25.4% and 26.4%, respectively). In contrast, patients receiving anidulafungin had the lowest mortality and therapeutic failure rates (4.8% and 5%) [154].

Candidemia and invasive candidiasis, specifically intra-abdominal candidiasis, are the predominant infectious syndromes of fungal etiology in intensive care units [155], mainly in patients with a history of abdominal surgery, where the duodenum and stomach have been identified as the site of origin of the infection (OR 4.188; 95% CI 1.204–14.561; *p* = 0.024 and OR 7.595; 95% CI 1.934–29.83; *p* = 0.004), respectively. The diagnosis of intra-abdominal candidiasis represents a challenge due to the difficulty of distinguishing between colonization and infection. Furthermore, culture-based methods exhibited poor diagnostic yields, with up to 6.9% being reported as positive in this context [156].

The niche for the development of antifungal drug resistance due to the emergence of resistant mutant strains lies in this clinical scenario. This phenomenon is explained by the patient’s severity, which generates changes in pharmacokinetic/pharmacodynamic (PK/PD) parameters, such as an increase in the volume of distribution and renal clearance, hypoalbuminemia, and the presence of abdominal drainage, as well as difficulty in adequately and effectively controlling the infection site. This particularly affects the efficacy of hydrophilic antifungals, such as echinocandins [157].

Echinocandins have a clinical effectiveness parameter based on AUC/MIC and Cmax/MIC. In this sense, some studies have quantified a concentration up to 33% lower in peritoneal fluid compared to serum concentrations [156]. After analyzing the maximum concentrations in peritoneal fluid in correlation with the clinical breakpoints according to CLSI and EUCAST, it is evident that the concentrations of echinocandins in peritoneal fluid are mostly insufficient for reaching the clinical effectiveness parameter, except in 10% of *C. albicans* species and in less than 1% for *N. glabratus*, *C. parapsilosis*, and *C. tropicalis*. Some optimization strategies proposed to achieve the clinical effectiveness parameter are dose adjustment in patients with obesity, as well as the use of amphotericin B deoxycholate, which is secondary to its concentration profile in peritoneal fluid relative to serum and greater effects on the biofilm [156].

### 7.8. Monitoring

The use of monitoring serum concentrations of antifungals such as posaconazole, voriconazole, and 5-fluocytocin are supported by robust evidence on their impact on clinical outcomes and toxicity [146]. The impact of other antifungals is under study. However, routine access to these drugs would limit their recommendation [158].

The use of echinocandins for the treatment of candidemia has increased, and prior treatment with this group of antifungals has been associated as a risk factor for therapeutic failure (OR 8.3; 95% CI 1.7–40.4; *p* < 0.01) [159]. Considering the pharmacological efficacy parameter and the administration dosage based on fixed doses and without adjustment to constitutional parameters, it may be related to the suboptimal exposure of the antifungal [160]. Particularly with micafungin, systemic clearance has shown a linear relationship with body weight, so in the case of overweight patients they could present a decrease in AUC and, consequently, a decrease in efficacy. In this regard, Nagamizu et al. [161] evaluated the relationship between micafungin dose and constitutional parameters (BMI, lean weight, and body surface area) and therapeutic success, defined by clinical and microbiological response. The results highlight that only an individualized dosage of micafungin based on body surface area ≥ 100 mg/m^2^ is significantly associated with a shorter time to therapeutic success (*p* =0.049). These results could imply a reduction in treatment time and, consequently, a reduction in the risk of treatment failure and the selection of resistant mutants. Therefore, patients with a surface area ≥ 100 mg/m^2^ could benefit from a dose of 200 mg/day of micafungin [158].

Similar results were found when evaluating the dosage of 200 mg of anidulafungin as an initial dose and subsequently 100 mg/day in patients with obesity defined by a BMI > 30 kg/m^2^. Patients with obesity had a statistically significantly longer time to microbiological cure, defined by the time to negative blood cultures, which was 4 days (3–6 days) vs. 3 days (2–5 days), *p* = 0.04 [162].

### 7.9. New Antifungal Drugs

The therapeutic options available for the treatment of fungal infections are limited, an alarming situation considering the incidence and mortality attributable to this cause. This limitation is further exacerbated by factors that increase resistance, such as climate change and the use of fungicides in the environment [160].

In the previous decade, rezafungin was the only novel drug approved for the treatment of candidemia and invasive candidiasis. It is a lipopeptide echinocandin that inhibits the formation of 1,3-β-D-glucan, an integral component of the fungal cell wall, by inhibiting β-D-glucan synthase [163]. This drug demonstrated an adequate safety and efficacy profile compared to caspofungin, with a weekly dosage of 400/200 mg, and demonstrated a significant advantage in reducing the time to negative blood cultures (19.5 h vs. 22.8 h, *p* = 0.04). Finally, its non-inferiority in overall and 90-day mortality was documented [163,164]. During clinical trials of this biologic, adverse effects were identified hypokalemia, pyrexia, diarrhea, anemia, vomiting, and hypomagnesemia. However, precautions for patients receiving rezafungin include infusion-related reactions, photosensitivity, and adverse hepatic reactions [163].

Non-inferiority results were maintained even when clinical outcomes (clinical response at day 14 of treatment, overall mortality rate, and mycological response at days 5 and 14 of treatment) were correlated with species of *Candida* and MICs (according to EUCAST Edef 7.4). However, a higher overall cure rate at 14 days was noted for *N. glabratus* (71% vs. 60%) and *C. parapsilosis* (78% vs. 55.6%) with rezafungin and caspofungin, respectively. Regarding mortality, this outcome was higher for *C. tropicalis* and *C. parapsilosis* with rezafungin and caspofungin (18% vs. 31%; 7.1% vs. 29.9%). Phenotypic susceptibility analysis documented three patients with infection with a strain that is non-susceptible to rezafungin. Of these patients, one in the rezafungin treatment group had the FKS mutation, but this was not associated with an impact on the overall clinical and mycological cure rate. In this regard, the multifactorial impact of candidemia treatment was demonstrated, which is not proportionally related to the MICs of the antifungal agent, and in turn, the benefit in the pharmacokinetic profile of the initial loading dose against species with high MICs is reinforced [165].

Additionally, considering the global care environment for patients with candidemia, it has been reported that up to 16% remain hospitalized to complete intravenous antifungal treatment for multiple reasons, such as fluconazole resistance (reported in up to 31%), tolerability, hepatotoxicity, and the benefit of the anti-biofilm activity of echinocandins. In this context, one in five patients with candidemia could benefit from treatment with rezafungin, thereby reducing care costs associated with prolonged hospitalization [11].

The results of the ReSPECT trial (NCT04368559) are currently pending publication. This is a phase III, multicenter, randomized, double-blind study that seeks to evaluate the efficacy and safety of prophylactic rezafungin administered as an injectable form in adult patients undergoing allogeneic bone marrow transplantation compared to standard therapy (fluconazole or posaconazole plus TMP-SMX). For this reason, its use in clinical practice, i.e., in real-life scenarios, is limited, as is its access.

Another promising antifungal is fosmanogepix, a prodrug of manogepix. It is active against yeasts, including *C. auris*, and some filamentous fungi such as *Fusarium* spp. It acts by inhibiting the *Gwt1* enzyme, which catalyzes the anchoring of mannoproteins to β-1,6-glucan, consequently affecting critical functions such as biofilm and germ tube formation. A phase II study published in 2023 evaluated its safety and efficacy as first-line treatment in adult patients with candidemia without neutropenia. Patients with deep-seated candidiasis and *P. kudriavzevii* infection were excluded. The results were promising for their impact on success rate, survival, time to negative blood cultures, transition to oral therapy, in vitro potency, and safety. The success rate at the end of the study was 80% and 30-day survival was 85%. Additionally, no serious adverse effects were directly attributable and no changes in serum concentration were documented in relation to pharmacokinetic alterations secondary to renal injury [18]. However, it is still pending approval by the FDA and EMA.

### 7.10. Other Considerations

In addition to antifungal treatment, other complementary therapeutic interventions are recommended due to their potential impact on mortality and disease progression. However, some of these strategies remain controversial due to limited evidence or conflicting results. This controversy highlights the need for future research designed to overcome these limitations and define their efficacy and impact on treatment.

### 7.11. Removal of Vascular Access in Patients Without Neutropenia

Vascular access removal in patients without neutropenia and with suspected vascular access origin is recommended by international management guidelines [121,123,146]. Although outcomes in patients with access removal are controversial, the beneficial effect warrants access removal whenever possible. However, with the aim of improving outcomes and mortality, the optimal time for removal has not yet been defined. The most recent update of the management consensus emphasizes that removal should be performed as soon as possible, suggesting a time limit of <48–72 h [146].

Several studies have been conducted to define the optimal timing of vascular access withdrawal, which impacts clinical success, as well as persistence, recurrence, microbiological elimination, and mortality. Nucci et al. [166] found that early withdrawal, defined within the first 24–48 h after the start of treatment, was associated with an increased therapeutic success rate and survival at 28 and 42 days. However, after adjusting these outcomes for severity of disease, persistence of neutropenia, and age, the association was not maintained. These findings highlight that both therapeutic success and mortality are primarily influenced by factors and the patient’s severity of disease. Comparable results confirmed the findings when evaluating 285 patients with candidemia and the impact of early vascular access withdrawal, defined within 48 h of diagnosis, compared to late withdrawal (>48 h) and withdrawal at any time. The overall mortality rate was 57.9%, with a mortality rate of 93.8% for patients without access removal, and 55.7% and 40% for early and late access removal, respectively. Multivariate analysis documented elevated APACHE II score (OR 1.11; 95% CI 1.066–1.15, *p* < 0.001), C parapsilosis (OR 0.291; 0.133–0.638, *p* < 0.002), and access removal at any time (OR 0.079; 0.021–0.298, *p* < 0.001) as risk factors associated with 30-day mortality [167]. This emphasizes the impact of maintaining vascular access on mortality and, in turn, considers the decreased risk of mortality in patients with access removal at any time as a direct effect of the patient’s severity. From this perspective, it is suggested to individualize and rationalize the withdrawal of access, prioritizing the patient’s severity and plausibility.

In patients with candidemia, due to their severity, it is necessary to maintain vascular access for medication administration and monitoring. In most patients in whom removal is successful, the timing of the placement of a new access point has been linked to the risk of recurrence, considering that the surface of the vascular access provides an adhesion site for biofilm production, promoting immune evasion, persistence, and consequently, recurrence. To evaluate the impact of the timing of new access placement on clinical outcomes such as incidence of persistence, mortality, and complications, Lee et al. [168] reported in a retrospective study that mortality was directly related to the etiology of *Candida* spp., without identifying that delayed access insertion (>3 days after removal) was associated with recurrence and mortality, probably due to the small number of patients in whom placement was delayed, again highlighting the severity of the patients and the need to ensure vascular access [168].

To optimize treatment in patients for whom vascular access removal is not possible, pharmacological strategies have been proposed that prioritize PK/PD parameters that impact biofilm reduction. Among these, the biofilm-reducing effect has been demonstrated primarily with echinocandins, primarily micafungin, and liposomal amphotericin [169].

### 7.12. Ophthalmological Evaluation

The ECMM, IDSA, and Australian consensus guidelines recommend ophthalmologic examination in all non-neutropenic patients during the first week of antifungal treatment. In patients with neutropenia, this evaluation should be performed once hematologic recovery has been documented [18,123].

In contrast, the American Academy of Ophthalmology (AAO) does not recommend generalized ophthalmologic evaluation in asymptomatic patients [170,171]. This position is based on the low prevalence of ophthalmologic involvement (<1%), according to a systematic review that included 952 prospectively evaluated patients older than 12 years without neutropenia. Furthermore, limitations in case definition, potential selection bias, and a lack of evidence regarding the impact of this intervention on long-term outcomes are noted.

These findings contrast with the results of Phongkhun et al. [172], who, using a standardized diagnostic framework for concordant ocular candidiasis and endophthalmitis and excluding studies with a substantial risk of bias, reported a pooled prevalence of 12.53% and 11.81%, respectively. In the subgroup analysis, a higher prevalence of endophthalmitis was documented in the Asian population (3.64%) compared to the European (1.40%) and American (1.44%) populations (*p* < 0.01) (56). Among the risk factors that have been significantly associated with the diagnosis of endophthalmitis are the administration of total parenteral nutrition (pOR: 3.02; 95% CI: 1.67–5.46; *p* < 0.01) and *C. albicans* infection (pOR: 6.92; 95% CI: 3.58–13.36; *p* < 0.01) (56). Similarly, a retrospective study that evaluated the prevalence, ophthalmological evaluation rate, risk factors, and outcomes in patients with candidemia estimated a necessary index of 20 patients to be screened to identify one patient with ocular candidiasis [173].

Considering symptom-directed screening, Shin et al. [174] reported that at least 48% of patients diagnosed with endophthalmitis were asymptomatic. Another study documented similar findings, with 44% of patients being asymptomatic, and in 22% it was not possible to document the presence of symptoms due to critical condition and sedation. This situation highlights the indication for ophthalmological evaluation, considering the high incidence in certain clinical settings, such as intensive care units, which account for up to 60% of cases, followed by oncology and transplant units, with around 13% of diagnoses [175]. These data reinforce the need to consider the clinical setting, risk factors, and additional microbiological findings to guide screening strategies, considering that not performing a screening could have consequences detrimental to survival and increased mortality (HR 2.19; 95% CI 1.55–3.11; *p* < 0.0001), as reported by Hoenigl et al. [131].

Regarding the timing of ophthalmologic evaluation in patients without neutropenia, a higher likelihood of detecting chorioretinitis has been documented when the ophthalmologic examination is performed after the seventh day following the blood culture result (3.6% vs. 14.3%; *p* = 0.35), in contrast to current recommendations. However, the decision to delay evaluation should be carefully evaluated, considering the prognostic and treatment implications of not identifying ocular involvement in a timely manner. The prognosis of these patients is closely related to the promptness of diagnosis and the timely initiation of targeted therapy [176].

### 7.13. Assessment by an Infectious Disease/Microbiology Specialist

As has been documented in other infectious syndromes with high morbidity and mortality, the intervention and follow-up of a specialized team positively influences the outcomes in patients with candidemia [177,178,179,180]. With this approach, Kobayashi et al. [178] identified that patients evaluated by an infectious disease specialist had significantly lower mortality at 30 and 90 days of 18% and 23%, compared to 50% in those who were not. Interestingly, the data reported by Hoenigl et al. [131] also demonstrated that the effect on mortality reduction was related to early intervention, that is, within the first 24–48 h, with a mortality reduction of 42% (HR 0.58; 95% CI; 0.44–0.70, *p* < 0 0001).

Finally, as has been demonstrated after analyzing the currently available therapeutic strategies, as well as complementary actions for the control of the infection site and follow-up of patients with candidemia, none are independently sufficient for eliminating excess mortality. This can be concluded from the evaluation of adherence to international recommendations, showing that in patients who did not receive these treatments, and after adjusting for initial severity and comorbidities, an increase in the mortality rate was observed, which reached between 51% and 71%, compared to the overall rate of 46% recorded in the total population evaluated [131].

Therefore, the importance of adhering to the recommendations for the diagnosis and treatment of candidemia is highlighted, but we advocate an individualized approach and treatment based on the risk factors and clinical characteristics of the patient, efficiently using the available diagnostic tools, based on the local epidemiological and microbiological context, and optimizing antifungal treatment according to PK/PD parameters in accordance with the severity and site of origin of the candidemia.

## 8. Conclusions

Currently, *C. albicans* is the main etiologic agent of candidemia worldwide; however, an increase in the isolation of rare or emerging species has been observed, such as *C. palmioleophilia*, *C. pelliculosa*, *C. rugosa*, *C. lusitaniae* (*Clavispora lusitaniae*), *C. digboiensis*, *C. utilis*, *C. innominate*, *C. ciferri*, *C. duobushaemulonii*, *C. kefyr*, *C. norvegensis*, *C. intermedia*, *C. pararugosa* (*W. pararugosa*), and *C. auris*. These species vary in susceptibility to antifungals, particularly when azoles are used, while echinocandins, as well as 5-fluorocytosine, are the best therapeutic alternative. Among the main risk factors, host factors such as immunosuppression and microbiota, chronic diseases, and age, in addition to the use of broad-spectrum antibiotics, the presence of a central venous catheter, parenteral nutrition, development of biofilms, and abdominal surgery, all contribute to the development of candidemia.

Of the diagnostic tools, innovative assays and modifications to existing ones have yielded satisfactory results in different patient populations. It is necessary to evaluate the context of candidemia development to apply the best diagnostic tool.

Finally, regarding antifungal treatment, due to the considerable risk in the neonatal population, in patients with prolonged neutropenia, and in critically ill patients undergoing abdominal surgery, antifungal prophylaxis is required to prevent the development of candidemia. During the establishment of antifungal therapy, monitoring is necessary to assess the impact on patient outcomes. Despite the approval of new antifungal treatments, such as rezafungin and fosmanogepix, it is necessary to evaluate the susceptibility patterns of clinical isolates of *Candida* spp. to reduce antifungal resistance.

## Figures and Tables

**Table 1:** Main mechanisms of resistance to antifungals in *Candida* spp.

Resistance Mechanism	Type of Antifungal	Functional Basis of Resistance	Example	References
Over-expression of membrane transporters: ABC (Adenosine Triphosphate (ATP)-Binding Cassette) transporters are encoded by the *CDR* (*Candida Drug Resistance*) genes, and MF (Major Facilitator) transporters by the *MDR* (*Multidrug Resistance*) genes. Over-expression of ABC transporters is the most common cause of resistance to all azoles, while over-expression of MF only confers resistance to fluconazole.	Azoles	Alterations in azole transport, overexpression of ABC and MF transporters increase the cell’s ability to eliminate azoles, which normally enter by passive diffusion, reducing their intracellular concentration and, therefore, decreasing their effectiveness.	Overexpression of *Cdr1p* and *Cdr2p* in *C. albicans*; *CgCdr1p* in *C. glabrata*; and *Cdr1p* in *C. auris*.	[66,67]
Mutations and/or overexpression of genes involved in ergosterol biosynthesis: *ERG11* is a gene that encodes lanosterol 14α-demethylase, which is crucial for the synthesis of ergosterol from the fungal cell membrane. Several point mutations have been identified that are associated with azole resistance. *ERG3* is another gene that also participates in the ergosterol pathway, converting episterol to ergosta-5,7,24(28)-trienol.	Azoles	Mutations in the *ERG11* sequence can affect the structure and function of lanosterol 14α-demethylase, reducing its affinity for azoles and decreasing the drug’s efficacy. Mutations in *ERG3* inhibit ergosterol biosynthesis from episterol to ergosta-5,7,24(28)-trienol.	The A61V, A114S, Y132F, Y132H, and I471T mutations in *ERG11* are associated with azole resistance in *C. albicans*; C108G, C423T, and A1581G in *C. glabrata*; A497C and G1570A in *C. krusei*. The Q139A mutations in *ERG3* cause azole resistance in *C. glabrata.*	[66]
Sterol import: It has recently been reported that sterol import may be a resistance mechanism.	Azoles	The reduction in ergosterol levels can be compensated by the exogenous import of sterols through specific importers and under anaerobic and microaerophilic conditions.	*C. glabrata* imports sterols using the importers Aus1p and Pdr11p, leading to increased resistance to azoles.	[66]
Genomic plasticity: loss of heterozygosity and aneuploidy are genomic variations that can lead to azole resistance.	Azoles	If one allele of a gene is mutated, loss of heterozygosity can copy the mutation to the second allele, causing loss of gene function.	Loss of heterozygosity in CaTAC1, CaERG11 and CaMRR1 of *C. albicans* has been correlated with increased azole resistance.	[66]
Alterations in the *ERG11* and ERG3 genes (lanosterol 14α-demethylase and sterol C-5 desaturase, respectively).	Polyenes	The loss of function of the *ERG11* and *ERG3* genes due to mutations alters the sterol composition of the cell membrane by the exchange of ergosterol for alternative sterols.	Loss of function of the *ERG11* and *ERG3* genes leads to the exchange of ergosterol for lanosterol, eburicol, and 4,14-dimethyl-zymosterol in the *C. albicans* membrane.	[68]
Alterations in the cell wall.	Polyenes	The increase in the 1,3-α-glucan fraction can physically inhibit the penetration of AmB through the cell wall.	An enlarged cell wall with increased levels of 1,3-β-glucan causes resistance to amphotericin B in *C. tropicalis*.	[69]
Point mutations in *FKS1* and *FKS2*, which encode β-(1-3) D glucan synthase, an important enzyme for cell wall biosynthesis.	Echinocandins	Mutations in *FKS1* and *FKS2* modify the target site in β-(1-3) D glucan synthase, preventing the action of echinocandins.	In *C. albicans*, *C. glabrata*, and *C. krusei*, it has been observed that the mutation in *FKS1* causes the substitution of serine 645 by proline, phenylalanine, and tyrosine, which modifies the target site and inhibits the binding of echinocandins to β-(1-3) D glucan synthase.	[67]
Mutation in the *FCY1* and *FCY2* genes, which encode the enzymes cytosine permease or cytosine deaminase which facilitate the absorption of fluorocytosine and its deamination, respectively.	5-FC	Mutations in *FCY1* and *FCY2* lead to inactivation of the enzymes they encode and cause decreased uptake or metabolic transformation of 5-FC and, therefore, resistance to the drug.	In *C. albicans*, inactivation of cytosine permease and cytosine deaminase has been shown to be associated with resistance to 5-FC.	[70]

**Table 2:** Main diagnostic methods for the identification of *Candida* spp.

Diagnostic Method	Sample/Target/Test	Advantages and Limitations	References
Blood culture	Peripheral blood	Easy to execute Identification of 21–71% at the gender level 72–96 h to obtain a result	[99]
Blood culture—Staining with calcofluor white	Peripheral blood	Staining of blood samples. Sensitivity of 90% Negative predictive value of 82% Recognition of *C. albicans* at a concentration of 10^5^–10^7^	[102]
1,3 β-D-Glucan	Peripheral blood	Sensitivity of 92% Specificity of 81%	[120]
PCR	AurisID^®^, Fungiplex^®^ *Candida auris*	Identification of *C. auris*	[106]
	CandID^®^, Fungiplex^®^ *Candida*, Fungiplex^®^ Universal, MycoReal *Candida* and MagicplexTM Sepsis	*C. albicans* *C. glabrata* *C. parapsilosis* *C. krusei* *C. dubliniensis* *C. tropicalis*	[106]
RAPD-PCR	CDC3 and HIS3	Allows the identification of six and seven alleles Evaluation of *C. albicans* isolates	[105]
Microarrays	Hybcell Pathogens DNA xB, SepsiTestTM-UMD and MycoReal Fungi, 28 S, 18S e ITS	Rapid tests that allow the establishment of prompt antifungal therapy They have not been validated for the diagnosis of invasive candidiasis	[106]
MALDI-TOF	Culture and peripheral blood	Accurate and rapid identification by performing this analysis in just 15 min No need to utilize expensive reagents or highly specialist equipment Cost of equipment is a main limitation for laboratories in developing countries	[16,99,115,116]
Next-generation sequencing	STI Region Dominino D1-D2	Inability to differentiate closely related species It is time-consuming and requires highly qualified personnel	[99]
T2 *Candida*	STI Region 5.8S and 28S rRNA	Identifies *C. albicans, C. tropicalis, C. parapsilosis, C. krusei*, and *C. glabrata* From blood and obtaining the result, an average time of <5 h Sensitivity of 65–100% Specificity of 90–100%	[110]
Biosensors	Electromechanical impedance, piezoelectric immunosensors, optical biosensor, loop-mediated isothermal amplification (LAMP), lateral flow strip, particle-mediated immunosensors, genosensors, fluorescence and flow cytometry, electromechanics, and microfluidic hydrodynamic cell trapping and epifluorescence	Fast, accurate, and cost-effective method Identifies *C. parapsilosis, C. albicans, C. krusei, C. tropicalis, C. glabrata*, and *C. auris* Sensitivities from 10^6^ cells/mL, up to 1 fg	[122]

## Supplementary Materials

The following supporting information can be downloaded at: https://www.mdpi.com/article/10.3390/pathogens14080806/s1. Table S1: Distribution and antifungal susceptibility of *Candida* species causing candidemia in the world, 2020–2025, [7,8,9,10,11,12,13,14,15,16,17,18,19,20,21,22,23,24,25,26,27,28,29,30,31,32,33,34,35,36,37,38,39,40,41,42,43,44,45,46,47,49,50,51,55,56,57,58,59,60,61,62,63,64,65,181,182,183,184,185,186,187,188,189,190].
