# Supplementary material for: Candidemia: An Update on Epidemiology, Risk Factors, Diagnosis, Susceptibility, and Treatment
Source: Pathogens. 2025 Aug 14;14(8):806. doi: 10.3390/pathogens14080806 (PMC12389578; doi:10.3390/pathogens14080806)
Supplement: Supplementary file 1 [file pathogens-14-00806-s001.zip › pathogens-3781097-supplementary.pdf]

Supplementary Table S1. Distribution and antifungal susceptibility of *Candida* species causing candidemia in the world, 2020-2025.

Species (%)	Country	Antifungal susceptibility MIC (µg/mL)	Resistance rate	Reference
		**AMERICA**		
		**Latin America**		
C. albicans (36.8), *C. parapsilosis* (22.8), *C. tropicalis* (21.0), *C. glabrata* (7.0), *C. lusitaniae* (5.3), *C. guilliermondii* (3.5), *C. auris* (1.8), *C. krusei* (0.9), *C. intermedia* (0.9)	Colombia		Higher resistance rate to FLZ in *C. parapsilosis*, followed by *C. glabrata*; and to AMB in *C. lusitaniae*, followed by *C. parapsilosis*	[7]
C. parapsilosis (100)	Mexico	FLZ (8-32)<br>VRZ (0.25-1)		[23]
C. auris (100)	Mexico		Resistance to FLZ was widespread	[28]
C. auris (100)	Peru	FLZ (≥512)<br>VRZ 0.12-0.25)<br>ITZ (0.12)<br>POS (0.06)<br>CAS (0.012-0.12)<br>ANF (0.25)<br>MIC (0.012-0.12)<br>5-FC (0.12-0.25)		[65]
		**North America**		
C. auris (100)	USA	AMB (1)<br>ANF (0.5-1)<br>MIC (0.12-0.25)<br>FLZ (>128)<br>MGX (0.008-0.015)<br>VRZ (1-2)		[29]
C. krusei (100)	Canada	MIC (0.12)<br>VRZ (1)<br>AMB (1)		[63]

		POS (0.5)		
C. nivariensis (100)	USA	5-FC (0.25)<br>AMB (1)<br>FLZ (4)<br>ITZ (0.5)<br>ISA (0.125)		[30]
C. auris (100)	USA	ANF (≤0.016-0.5)<br>CAS (0.06-≥16)<br>MIC (0.016-1)<br>FLZ (0.25-≥512)<br>ITZ 0(0.03-≥32)<br>POS (0.016-≥16)<br>VRZ (≤0.008-≥16)<br>AMB (0.25-4)<br>5-FC (≤0.06-16)		[24]
C. tropicalis (100)	USA	MIC (0.016)<br>FLZ (≥256)<br>ITZ (≥16)<br>VRZ (≥8)<br>POS (≥8)		[64]
C. auris (100)	USA	FLZ (≥256)<br>CAS (0.12)<br>5-FC (≥64)<br>AMB (1)		[31]
C. glabrata (45.4)<br>*C. albicans* (36.4)<br>*C. parapsilosis* (13.6)<br>*C. dubliniensis* (4.5)	USA, Israel, Belgium, Spain	ANF (0.016-4)<br>FLZ (0.25-4)<br>AMB (0.5-4)<br>MGX (0.008-0.03)		[18]
C. auris (100)	USA	MIC (<4)		[32]
C. dubliniensis (100)	USA		Sensitive to MIC and FLZ	[181]
C. glabrata (100)	USA		MIC sensitive	[25]
		**AFRICA**		
C. albicans (35), *C. parapsilosis* (29.7), *C. auris* (14.1), *C. glabrata* (4.7), *C. pelliculosa* (3.1), *C.*	Kenya, South Africa, Uganda, Bangladesh, Brazil, China,		*C. albicans* showed 91% susceptibility to FLZ and 100% to AMB. *C. parapsilosis* showed 59% resistance to	[8]

tropicalis (3.1), *C. famata* (0.8), *C. metapsilosis* (0.8), *C. rugosa* (0.8%), *Candida* spp. (7.8)	Greece, India, Italy, Thailand, Vietnam		FLZ and 93% susceptibility to AMB. *C. auris* presented high resistance to FLZ (88%), AMB (85%), and VRZ (31%). All *Candida* spp. isolates were susceptible to the three commonly used antifungals.	
C. parapsilosis (42), *C. albicans* (36), *Candida* spp. (22)	South Africa	FLZ (≥ 32) *C. parapsilosis*		[19]
		**OCEANIA**		
C. albicans (50), *C. glabrata* (50)	Australia	*C. albicans*: FLZ (< 0.05)<br>*C. glabrata*: FLZ (128)<br>CAS (0.06)<br>5-FC (≤0.06)<br>AMB (0.5)		[26]
C. duobushaemulonii (100)	Australia	FLZ (64)<br>MIC (0.06)<br>AMB (0.5)		[33]
		**EUROPE**		
		**Southern Europe**		
C. gulliermondii (100)	Portugal		Susceptible to AMB	[182]
C. albicans (51), *C. parapsilosis* (25), *C. glabrata* (10), *C. tropicalis* (9), *C. guilliermondii* (2), *C. krusei* (1), *C. lusitaniae* (1), *C. dubliniensis* (0.5), *C. kefyr* (0.1), *C. norvegensis* (0.1), *C. pelliculosa* (0.1), *C. rugosa* (0.1), *C. utilis* (0.1)	Italy	AMB (0.06-2)<br>FLZ (≤0.012->256)<br>VRC (≤0.008->8)<br>CAS (≤0.008-2)<br>ANF (≤0.015-4)<br>MIC (≤0.008-4)		[9]
C. albicans (33.1), *C. parapsilosis* (26.3), *C. glabrata* (12.0), *C. krusei*	Italy	AMB (≤0.12–2)<br>FLZ (≤0.12–256)<br>ITZ (≤0.015–32)<br>POS (≤0.008–16)		[10]

(10.5), *C. tropicalis* (9.8), *Candida* spp. (8.3)		VRZ (≤0.008–8)<br>ANF (≤0.015–8)<br>CAS (0.015-1)<br>MIC (≤0.008-2)		
C. auris (100)	Greece	AMB (0.5-1)<br>ANF (0.12-0.5)<br>MIC (0.12-0.5)<br>CAS (0.06-1)<br>ISA (0.015-0.06)<br>POS (0.03-0.5)<br>VRZ (0.5-8)<br>ITZ (0.12-0.5)<br>FLZ (128->256)		[34]
C. auris (100)	Italy	AMB (0.5)<br>CAS (0.125)<br>MIC (0.06)<br>VRZ (0.06)<br>ANF (0.03)<br>5-FC (32)<br>FLZ (>32)<br>ITZ (>4)<br>POS (≥8)		[35]
C. auris (100)	Italy	AMB (2-4)<br>ANF (0.12)<br>CAS (0.12)<br>FLZ (128-256)<br>ISA (0.06-0.12)<br>ITZ (0.12-0.25)<br>MIC (0.12)<br>VRZ (0.5)<br>POS (0.06)		[36]
C. parapsilosis (100)	Italy	FLZ (≥ 32)		[183]
C. albicans (100)	Italy	ANF (1)<br>MIC (4)<br>CAS (8)<br>FLZ (0.25)		[60]

		ITZ (0.06)<br>VRZ (<0.008)<br>5-FC (<0.06)<br>AMB (0.5)<br>POS (0.03)		
C. auris (100)	Spain		Sensitive to echinocandins	[37]
C. tropicalis (100)	Italy	CAS (0.016)<br>FLZ (>256)<br>VRZ (>8)<br>ANF (0.03)<br>REZ N/A<br>MIC (0.03)<br>5-FC (<0.06)<br>POS (>8)<br>AMB (0.5)<br>ISA (0.06)		[59]
C. parapsilosis (100)	Portugal		Sensitive to 5-FC, AMB, FLZ and VRZ, resistant to CAS	[61]
C. lusitaniae (100)	Greece	AMB (1-2)<br>FLZ (<0.125)<br>ITZ (0.125)<br>VRZ (≤0.016)<br>POS (≤0.016)<br>ANF (0.03-0.06)<br>CAS (0.5-1)		[38]
C. albicans (42), *C. glabrata* (31), *C. parapsilosis* (6), *C. tropicalis* (6), *C. krusei* (4), *C. auris* (3), *C. dubliniensis* (1), *C. lusitaniae* (1), *C. rugosa* (1), *C. digboiensis* (1), *Candida* spp. (4)	Austria, Germany, Serbia, France, Slovenia, Russia, Italy, Sweden, The Netherlands, Turkey, UK, Spain, Ireland, Belgium, Denmark		18% de resistencia a FLZ	[11]

**Eastern Europe**				
C. orthopsilosis (100)	Poland	AMB (0.25)<br>FLZ (0.19)<br>CAS (0.50)<br>MIC (0.75)<br>ANF (0.25)		[39]
C. glabrata (100)	Slovakia	MIC (0.015)<br>AMB (0.5)<br>ANF (0.125)<br>CAS (0.125)<br>FLZ (64)<br>VRZ (0.5)		[184]
**Western Europe**				
C. krusei (100)	The Netherlands	AMB (0.5)<br>ANF (0.023)<br>VRZ (0.5)		[185]
C. dubliniensis (100)	Belgium		Sensitive to ANF	[186]
C. tropicalis (100)	The Netherlands	FLZ (0.125-64)<br>VRZ (0.016-4)<br>ITZ (0.06-16)<br>MIC (0.008-1)<br>ANF (0.008-0.5)<br>AMB (0.125-2)		[62]
C. albicans and *C. glabrata* (100)	Germany	*C. glabrata*: ANF (0.25) CAS (0.25)<br>MIC (0.06)	*C. albicans* sensitive to all antifungals	[27]
**ASIA**				
**East Asia**				
C. albicans (28.0), *C. tropicalis* (27.3), *C. glabrata* (19.7), *C. parapsilosis* (13.6), Otras (*C. guilliermondii*, *C. pelliculosa*, *C. utilis*, *C.*	China		*C. albicans* showed 91% susceptibility to FLZ and VRZ. *C. glabrata* showed greater susceptibility to VRZ than to FLZ and ITZ. *C. tropicalis* showed 75% susceptibility to FLZ and	[12]

krusei, *C. lusitaniae*, and *C. innominate*) (11.4)			VRZ, and 61.1% to ITZ. All *Candida* species showed 100% susceptibility to AMB.	
C. auris (100)	Taiwan	FLZ (8-16)<br>AMB (0.5-1)<br>ANF (0.12-1)<br>MIC (0.06-1)<br>5-FC (≤0.06)<br>VRZ (0.12-0.25)<br>POS (0.12-0.25)<br>ISA (0.06-0.25)		[40]
C. albicans (39.4), *C. glabrata* (20.2), *C. tropicalis* (19.3), *C. parapsilosis* (15.6), *C. krusei* (2.3) and others (3.2)	China		All isolates showed high susceptibility to AMB (99.5%) and 5-FC (99%). The resistance rates to ITZ, VRZ, and FLZ were 18.6%, 15.2%, and 14.1%, respectively. *C. tropicalis* showed the highest resistance rates to FLZ (36.6%), ITZ (36.6%), and VRZ (39.0%).	[49]
C. auris (100)	Japan	FLZ (32)<br>VRZ (0.5)<br>AMB (0.5)<br>CAS (0.25)<br>MIC (0.06)		[41]
C. albicans (33.7), *C. glabrata* (11), *C. tropicalis* (8.1), *C. famata* (1.7), *C. guilliermondii* (1.2), *C. krusei* (0.6%), *Candida* spp. (0.6)	China	FLZ (≤0.5–32)<br>VRZ (≤0.03–4)<br>ITZ (≤0.062–8)<br>AMB (≤0.25–1)		[13]
C. albicans (43.6), *C. glabrata* (19.5), *C. parapsilosis* (18.8), *C. tropicalis* (6.7), *C. krusei*	Japón		*C. albicans* and *C. parapsilosis* < 6% resistance to FLZ, MIC, VRZ, AMB. *C. glabrata* resistant to FLZ < 9%, to MIC	[14]

(1.4), *C. guilliermondii* (1.7), *C. dublinensis* (0.15), and *Candida* spp. (8.1)			17.8%. *C. tropicalis* 17.8% resistant to FLZ and 13.5% to VRZ. *C. krusei* 35.7% resistant to AMB	
C. albicans (40.3), *C. glabrata* (36.3), *C. tropicalis* (13.9), *C. parapsilosis* (4), *C. krusei* (3), *Candida* spp. (2.5)	China		99% susceptibility to AMB and 5-FC. Resistance rates to ITZ, VRZ, and FLZ were 24.9%, 19.4%, and 18.5%, respectively. *C. tropicalis* had resistance rates of 39.3%, 39.3%, and 42.9% to FLZ, ITZ, and VRZ, respectively.	[50]
C. pararugosa (*Wickerhamiella pararugosa*) (100)	Japan	FLZ (4-8)<br>MIC (0.06)		[42]
C. tropicalis (100)	China	AMB (≤0.5)<br>FLZ (1)<br>VRZ (0.125)<br>5-FC (≤0.4)		[187]
C. auris (100)	China	FLZ (128)<br>CAS (8)<br>AMB (2)<br>ITZ (0.12)<br>VRZ (0.5)<br>ANF (0.5)<br>MIC (0.25)<br>5-FC (0.12)		[43]
C. guilliermondii (71)<br>*C. haemulonii* (9.1)<br>*C. lipolytica* (5.4)<br>*C. norvegensis* (5.4)<br>*C. pelliculosa* (5.4)<br>*C. lusitaniae* (3.6)	Taiwan	AMB (0.06->8)<br>5-FC (0.06->64)<br>FLZ (0.06->256)<br>ITZ (0.008-1)<br>POS (0.004->8)<br>VRZ (0.004->8)<br>ANF (0.008->8)<br>CAS (0.004->8)<br>MIC (0.004->8)		[20]
		**West Asia**		

C. palmioleophila	Iran	AMB (2)<br>FLZ (16)<br>ITZ (0.25)<br>VRZ (0.0625)<br>ISA (0.125)<br>POS (0.25)<br>NYS (4)<br>CAS (0.031)		[44]
C. albicans (47.9), non-*albicans Candida* (52.1)	Turkey		Resistance to FLZ was 20% (24.2% for *C. albicans* and 16.2% for NAC) and resistance to VRZ was 5.7% (12.1% for *C. albicans* and 0% for NAC)	[21]
C. albicans (100)	Turkey	AMB (0.125)<br>FLZ (1.00)<br>VRZ (0.032)<br>CAS (0.250)		[188]
C. duobushaemulonii (100)	Lebanon	AMB (8)<br>5-FC (≤ 1)<br>VRZ (4)		[45]
C. albicans and *C. tropicalis* (>70)<br>*C. parapsilosis* (<30)	Iran, Austria, Turkey		*C. tropicalis, C. parapsilosis* and *C. albicans* presented 50%, 20% and 13.6% resistance to azoles	[57]
C. albicans (47.4), *C. parapsilosis* (26.6), *C. tropicalis* (9.6), *C. glabrata* (7.6), *Candida* spp. (8.8)	Turkey		The FLZ resistance rate was 13% in *C. parapsilosis*. No resistance to echinocandins was detected.	[15]<br>Doğan 2020
C. albicans (100)	Iran		Sensitive to CAS	[33]
C. auris (100)	Kuwait	FLZ (≥128)<br>VRZ (0.25-0.5)<br>ITZ (0.5-1)<br>POS (0.031-0.063)<br>ANF (0.031-1)<br>MIC (0.016-1)		[189]

		AMB (1)		
C. glabrata (100)	Turkey	FLZ (0.5-64)<br>VRZ (0.032-0.25)<br>ITZ (0.032-0.25)<br>MIC (0.016-0.12)<br>ANF (0.016-0.25)<br>AMB (0.12-2)		[58]
C. albicans (52.4), *C. glabrata* (24.2), *C. parapsilosis* (12.1), *C. tropicalis* (6.5), *C. dubliniensis* (4.7)	Iran	FLZ (≥ 32)		[16]
C. parapsilosis (100)	Turkey	FLZ (0.5-≥32)		[190]
C. auris (100)	Saudi Arabia		ANF resistant	[46]
C. auris (100)	Iran	FLZ (>256)		[47]
		**South Asia**		
C. albicans (10.2). *C. parapsilosis* (41.0). *C. ciferrii* (23.0), *C. auris* (7.7), *C. rugosa* (10.3), *C. lusitaniae* (2.6)	Bangladesh		33% resistance to FLZ and 5% to VRZ was observed	[22]
C. parapsilosis complex (100)	Pakistan	FLZ (8-256)		[51]
C. glabrata (100)	India	AMB (2)<br>5-FC (2)		[56]
C. tropicalis (44)<br>*C. albicans* (22)<br>*C. glabrata* (19)<br>*C. parapsilosis* (12)<br>*C. krusei* (3)	India		*C. albicans* 14% resistant to CAS<br>*Candida* spp. 5% resistant to AMB<br>*C. tropicalis and C. glabrata* 17-33% resistant to FLZ<br>*C. krusei* 100% resistant to FLZ	[55]
C. albicans (35)<br>*C. glabrata* (25)	Thailand	ANF (0.015-8)<br>MIC (0.008-4)		[17]

C. parapsilosis (30)	CAS (0.03-8)
C. orthopsilosis (10)	5-FC (0.06-64)
	POS (0.008-8)
	VRZ (0.008-8)
	ITZ (0.03-16)
	FLZ (0.05-256)
	AMB (0.12-4)
